# Unraveling Verapamil’s Multidimensional Role in Diabetes Therapy: From β-Cell Regeneration to Cholecystokinin Induction in Zebrafish and MIN6 Cell-Line Models

**DOI:** 10.3390/cells13110949

**Published:** 2024-05-30

**Authors:** Hossein Arefanian, Ashraf Al Madhoun, Fatema Al-Rashed, Fawaz Alzaid, Fatemah Bahman, Rasheeba Nizam, Mohammed Alhusayan, Sumi John, Sindhu Jacob, Michayla R. Williams, Nermeen Abukhalaf, Steve Shenouda, Shibu Joseph, Halemah AlSaeed, Shihab Kochumon, Anwar Mohammad, Lubaina Koti, Sardar Sindhu, Mohamed Abu-Farha, Jehad Abubaker, Thangavel Alphonse Thanaraj, Rasheed Ahmad, Fahd Al-Mulla

**Affiliations:** 1 Department of Immunology & Microbiology, Dasman Diabetes Institute, Dasman 15462, Kuwait; hossein.arefanian@dasmaninstitute.org (H.A.); fatema.alrashed@dasmaninstitute.org (F.A.-R.); fatemah.bahman@dasmaninstitute.org (F.B.); steve.shenouda@dasmaninstitute.org (S.S.); halemah.alsaeed@dasmaninstitute.org (H.A.); shihab.kochumon@dasmaninstitute.org (S.K.); sardar.sindhu@dasmaninstitute.org (S.S.); rasheed.ahmad@dasmaninstitute.org (R.A.); 2 Department of Genetics and Bioinformatics, Dasman Diabetes Institute, Dasman 15462, Kuwait; ashraf.madhoun@dasmaninstitute.org (A.A.M.); rasheeba.iqbal@dasmaninstitute.org (R.N.); sumi.john@dasmaninstitute.org (S.J.); sindhu.jacob@dasmaninstitute.org (S.J.); lubaina.koti@dasmaninstitute.org (L.K.); alphonse.thangavel@dasmaninstitute.org (T.A.T.); 3 Animal and Imaging Core Facility, Dasman Diabetes Institute, Dasman 15462, Kuwait; nermeen.abukhalaf@dasmaninstitute.org; 4 Department of Bioenergetics & Neurometabolism, Dasman Diabetes Institute, Dasman 15462, Kuwait; fawaz.alzaid@dasmaninstitute.org (F.A.); mohammed.alhusayan@dasmaninstitute.org (M.A.); michayla.williams@dasmaninstitute.org (M.R.W.); 5 Institut Necker Enfants Malades (INEM), French Institute of Health and Medical Research (INSERM), Immunity & Metabolism of Diabetes (IMMEDIAB), Université de Paris Cité, 75014 Paris, France; 6 Special Services Facilities, Dasman Diabetes Institute, Dasman 15462, Kuwait; shibu.joseph@dasmaninstitute.org; 7 Department of Biochemistry and Molecular Biology, Dasman Diabetes Institute, Dasman 15462, Kuwait; anwar.mohammad@dasmaninstitute.org (A.M.); mohamed.abufarha@dasmaninstitute.org (M.A.-F.); jehad.abubakr@dasmaninstitute.org (J.A.); 8 Department of Translational Research, Dasman Diabetes Institute, Dasman 15462, Kuwait

**Keywords:** β-cells, verapamil, calcium channel blocker, zebrafish, MIN6 cells, diabetes mellitus

## Abstract

This study unveils verapamil’s compelling cytoprotective and proliferative effects on pancreatic β-cells amidst diabetic stressors, spotlighting its unforeseen role in augmenting cholecystokinin (CCK) expression. Through rigorous investigations employing MIN6 β-cells and zebrafish models under type 1 and type 2 diabetic conditions, we demonstrate verapamil’s capacity to significantly boost β-cell proliferation, enhance glucose-stimulated insulin secretion, and fortify cellular resilience. A pivotal revelation of our research is verapamil’s induction of CCK, a peptide hormone known for its role in nutrient digestion and insulin secretion, which signifies a novel pathway through which verapamil exerts its therapeutic effects. Furthermore, our mechanistic insights reveal that verapamil orchestrates a broad spectrum of gene and protein expressions pivotal for β-cell survival and adaptation to immune-metabolic challenges. In vivo validation in a zebrafish larvae model confirms verapamil’s efficacy in fostering β-cell recovery post-metronidazole infliction. Collectively, our findings advocate for verapamil’s reevaluation as a multifaceted agent in diabetes therapy, highlighting its novel function in CCK upregulation alongside enhancing β-cell proliferation, glucose sensing, and oxidative respiration. This research enriches the therapeutic landscape, proposing verapamil not only as a cytoprotector but also as a promoter of β-cell regeneration, thereby offering fresh avenues for diabetes management strategies aimed at preserving and augmenting β-cell functionality.

## 1. Introduction

Consistently escalating morbidity and complications associated with diabetes have become a matter of growing concern. As of 2021, globally, 537 million (1 in 10) adults were living with diabetes, and these staggering figures are predicted to rise up to 783 million by 2045 [1]. Although in the past century, the discovery of insulin has improved the therapeutic options for patients with diabetes, certain limiting factors, such as insufficient efficacy, cost, weight gain, and life-threatening hypoglycemic episodes remain the prohibitive challenges that are commonly associated with the use of insulin in humans [2,3].

Type 2 diabetes mellitus (T2DM) is a chronic metabolic disorder, accounting for 90–95% of all diabetic cases. Its pathophysiology involves a combined effect of long-term insulin resistance and β-cell impairment and consequently, reduced insulin secretion and hyperglycemia, ultimately leading to pancreatic β-cell dysfunction [4]. Type 1 diabetes (T1D) is an autoimmune and inflammatory disease that is characterized by a loss of pancreatic β-cells. Its treatment comprises exogenous insulin to maintain normoglycemia, which requires continuous infusion or multiple daily injections, with no oral medications available. Moreover, treatment with exogenous insulin does not reverse the loss of β-cell mass and function [5]. Although therapeutic approaches aimed at improving insulin delivery and glucose uptake are necessary for the treatment of diabetes, they are not designed to target the disease at its root cause, i.e., β-cell dysfunction or loss. 

Verapamil is a calcium channel blocker (CCB) that was approved by the Food and Drug Administration (FDA) in the late 1990s for the treatment of hypertension, angina, and arrhythmia [6]. Notably, it was found to lower the risk of developing new-onset diabetes [7,8,9], as compared to other CCBs, and was also found to lower fasting glucose levels in patients with diabetes [3,10,11,12,13,14]. Verapamil is known to act by inhibiting the L-type calcium channels and reducing the cellular calcium influx, thereby inhibiting the transcription of the thioredoxin-interacting protein (TXNIP) [14,15,16], which would otherwise accumulate and increase oxidative stress-related toxicity in the pancreas [11,17,18]. While intracellular calcium is required to trigger insulin exocytosis, chronic increase in calcium levels may result in β-cell impairment [19]. This preventative mechanism of verapamil is highly attributed to TXNIP downregulation, which further leads to a decrease in β-cell apoptosis [14,15,19]. 

Verapamil ameliorated β-cell survival and activity, enhanced insulin levels, improved glucose sensitivity and homeostasis, and reduced hypoglycemic episodes in both mice and patients with T1D [3,11,13,14,15]. Oral verapamil administered to patients with T1D lowered dependency on exogenous insulin and improved endogenous β-cell function, measured using the C-peptide area under the curve (AUC), as compared to the placebo. Additionally, it decreased the frequency of hypoglycemic events [3,11,13,14,15]. Similarly, in mice, oral verapamil lowered TXNIP expression, increased endogenous insulin levels, and reduced β-cell apoptosis, rescuing the mice from streptozotocin (STZ)-induced diabetes [15,20]. In human clinical trials, verapamil showed a reduced risk of new-onset diabetes, as compared to atenolol use [8,9]. 

These interesting studies led us to further investigate the mechanisms by which verapamil renders beneficial effects in MIN6 mouse pancreatic β-cells and transgenic zebrafish models, specifically focusing on β-cells viability and function, after challenges with immune-metabolic stressors mimicking the T1D/T2D insults.

## 2. Materials and Methods

### 2.1. Cell Culture and Treatments

MIN6 cells, a mouse β-cell line, were generously gifted by Dr. Jun-ichi Miyazaki, Kumamoto University Medical School, Japan [21]. The cells were cultured and maintained in Dulbecco’s modified Eagle’s medium (DMEM), which contained 25.2 mM glucose, supplemented with 15% heat-inactivated fetal bovine serum (FBS), 2 mM l-glutamine, 1 mM sodium pyruvate, 10 mM HEPES, 50 μg/mL penicillin, 100 μg/mL streptomycin, and 70 mM β-mercaptoethanol at 37 °C, with 5% CO_2_ (all reagents were purchased from Invitrogen, Waltham, MA, USA). The cells were re-fed every 2 to 3 days [21]. For experimental assays, cells were maintained in the supplemented DMEM containing 5.6 mM glucose. All analyses using MIN6 cells were performed at a passage of <25. 

A stock solution of verapamil hydrochloride (152-11-4, Calbiochem, Burlington, MA, USA) was freshly prepared in phosphate-buffered saline (PBS). The cells were treated with verapamil at different concentrations, depending on each experimental setting. 

### 2.2. Cell Survival

Cells were seeded at a concentration of 5 × 10^4^ cells/well in a 96-well plate (Costar, High Wycombe, UK). Based on the experimental set-up, cells were treated with verapamil and/or different stressors (details included in Supplementary Methods), and cell survival was determined using an MTT (3-[4,5-dimethylthiazol-2-yl]-2,5 diphenyl tetrazolium bromide) assay (Trevigen, Minneapolis, MN, USA), following the manufacturer’s instructions. Plates were read using a Synergy H4 Hybrid Microplate Reader (BioTek, Winooski, VT, USA), and the data were analyzed using Gen5 software ver 2.03.

### 2.3. Cell Proliferation

MIN6 β-cell proliferation was determined by monitoring growth consecutively for 24 h. To perform proliferation assays, cells were seeded at a concentration of 5 × 10^4^ cells/well in 24-well plates (Costar, High Wycombe, UK) and incubated overnight at 37 °C in 5% CO_2_. Later, verapamil was added (50 μM) every 24 h for 7 days, followed by trypsinization, harvesting, and cell counting using a trypan blue dye exclusion assay.

### 2.4. Immunohistochemical Detection of Ki67

To test the effect of verapamil on MIN6 β-cell proliferation, immunohistochemical (IHC) analysis of Ki67 was performed, as described previously [22]. Briefly, cells were seeded on coverslips in media supplemented with 2% bovine serum albumin (BSA) free fatty acid (FFA) (Cat# 820024, Sigma, St. Louis, MO, USA) for 24 h. Then, cells were either treated with verapamil (50 μM) for another 24 h or left untreated to serve as control. Cells were stained using rabbit anti-mouse Ki67 primary antibody and goat anti-rabbit secondary antibody; actin filaments and nuclei were counterstained using Phalloidin-iFluor 594 reagent and a mounting medium with DAPI (details included in Supplementary Methods). Confocal images were obtained using an inverted Zeiss LSM710 spectral confocal microscope (Carl Zeiss, Gottingen, Germany). All samples were analyzed using the same parameters, and the resulting color markup for analysis of each sample was confirmed. Correlated total cell fluorescence (CTCF) was calculated from 10 different fields of each number (n) via ImageJ ver. 2.9.0, using the following equation: 

CTCF = Integrated Density − (Area of selected cell × Mean fluorescence of background readings) [23].

### 2.5. Western Blotting

MIN6 cells were seeded in 6-well plates (Corning, Somerville, MA, USA) and incubated in media supplemented with 2% BSA free-FFA for 24 h. Then, cells were either treated with verapamil (50 μM) and incubated for another 24 h or left untreated (control). Later, total protein was extracted, and Western blotting was performed as previously described [24,25] (details included in Supplementary Methods).

### 2.6. Cell Cycle Analysis

Cell cycle analysis was carried out using flow cytometry, based on propidium iodide (PI) staining of the DNA, as described before [26]. Briefly, MIN6 cells were seeded in 12-well plates (Costar, High Wycombe, UK) in media supplemented with FFA-free 2% BSA for 24 h. Then, cells were treated with verapamil (50 μM) for 1, 2, 3, or 4 h, or left untreated (control). Following trypsinization, re-suspended cells were fixed with ice-cold 70% ethanol, harvested by centrifugation (800 × *g* for 5 min), treated at room temperature with RNase (100 µg/mL) for 30 min, followed by incubation with PI (40 µg/mL) for another 30 min. DNA content and cell cycle were analyzed using a BD FACSCant Flow Cytometer (BD Bioscience, San Jose, CA, USA). Cells were gated and analyzed for doublet exclusion. A single cell population was acquired for analysis at 490 nm Ex and 630 nm Em wavelengths. Data were analyzed using BD FACSDivaTM Software 8 (BD Biosciences, San Jose, CA, USA).

### 2.7. Protection Assays

For protection assays, verapamil was used at concentrations of 1, 5, 10, and 50 μM. The protection assays were conducted for verapamil treatment in pretreatment, cotreatment, and pretreatment followed by cotreatment conditions, and challenged the cells with STZ and T1D-/T2D-cytomixes, as indicated elsewhere [27,28] (details included in Supplementary Methods).

### 2.8. Glucose-Stimulated Insulin Secretion (GSIS) Assay 

A GSIS assay was performed using the static incubation method, as described before [29] (details included in Supplementary Methods). Briefly, 1 × 10^5^ cells/well were seeded in 24-well plates (Corning, Somerville, MA, USA) and incubated for 24 h. Later, MIN6 cells were treated with verapamil (50 μM) for another 24 h. Then, the cells were incubated with Krebs–Ringer HEPES buffer supplemented with different concentrations of glucose at 37 °C. GSIS assay was terminated, the conditioned media were collected, and levels of secreted insulin were detected using ultrasensitive mouse insulin ELISA kit (Mercordia, Sylveniusgatan, Uppsala, Sweden). Total insulin content in cells was measured by adding cold acidified ethanol to each well and keeping it overnight at −80 °C. After three freeze/thaw cycles, cells were scraped off and supernatants were collected by centrifugation, neutralized with 1 M Tris pH 7.5, and insulin levels were measured using ELISA. The levels of total insulin content and secreted insulin were normalized by the level of total protein measured by Bradford assay against a standard curve.

### 2.9. Metabolic Flux Analysis (Seahorse Assay)

The oxygen consumption rate (OCR) was analyzed by a mitochondrial stress test using the manufacturer’s protocol (Seahorse XFe96, Agilent Technologies, Santa Clara, CA, USA). Briefly, MIN6 cells were seeded in Agilent Seahorse XF96 Cell Culture Microplate (Cat#: 101085-004) at a concentration of 4 × 10^4^ cells/well and incubated for 24 h, and later, cells were either treated with STZ (3 mM) and/or verapamil (50 μM) or were left untreated (control). At the end point of each condition, an OCR assay was performed using compounds in assay media (supplemented with 5.6 mM glucose) at the following concentrations: Oligomycin (1 µM), FCCP (2 µM), and Rot/AA (0.5 µM) (Cat# 103015-100; Agilent Technologies Seahorse XF Cell Mito Stress Test Kit, Santa Clara, CA, USA). The levels of basal and maximal respirations were calculated, and OCR was normalized to the extracted total protein as an indirect estimation of the number of cells [30]. 

### 2.10. Whole-Transcriptome Analysis

Whole-transcriptome analysis was conducted as previously described [25] (details included in Supplementary Methods). Briefly, total RNA was isolated from verapamil-treated (50 μM; 24 h) and untreated (control) MIN6 cells using a RNeasy kit (Qiagen, Hilden, Germany), following standard protocol. For whole-transcriptome sequencing, RNA (40 ng) was used to prepare the transcriptome libraries, using a Truseq stranded mRNA kit (Illumina Inc., San Diego, CA, USA), following the manufacturer’s protocol. The libraries were validated and quantified using a bioanalyzer (Agilent Technologies, Santa Clara, CA, USA) and qubit fluorometer (Thermofisher Scientific, Santa Clara, CA, USA), respectively. Paired-end sequencing was carried out using the Novaseq 6000 system (Illumina Inc. San Diego, CA, USA), and the BCL files were converted to Fastq using the bcl2fastq ver. 2.20 tool. Quality control of Fastq files was performed using FastQC (ver. 0.11.9). Trimmomatic (ver. 0.39) was used for the adaptor and quality trimming, and for removing extremely short reads, and HISAT2 (ver. 2.1.0) was used for data alignment. To enumerate the number of reads associated with the genes, the htseq-count tool was used in HTSeq (ver. 0.9.1). Differential gene expression analysis was performed using the Bioconductor package edgeR, using the default setting. The presented data are from four sets of independent experiments, and from each set, three replicates were processed to control for intra- and inter-assay errors.

### 2.11. Proteome Sample Preparation 

Proteome sample preparation was conducted based on the standard protocol [31] (details included in Supplementary Methods). Briefly, frozen pellets of MIN6 cells treated with verapamil (50 μM; 24 h) and untreated cells (control) were lysed, reduced using reduction buffer at 37 °C, and then digested by incubating with trypsin at 37 °C overnight. Next, the samples were acidified and cleaned by using C-18 MACROSpin plates, and the digested peptides were eluted in 340 µL/well of C18 elution solution (0.1% *v*/*v*) TFA in 50% acetonitrile. Samples were dried, reconstituted (1 µg/µL), and analyzed using Q-Exactive liquid chromatography-electrospray ionization-tandem mass spectrometry (LC-ESI-MS/MS) coupled with an EASY-nLC™ 1200 nano-LC System, through an EASY-Spray Ion Source (Thermo Fisher Scientific, Waltham, MA, USA). Peptides were eluted and separated using a 170 min run, and the eluent was ionized using Easy Spray nano ESI source operating in positive ion mode. Label-free quantitation was performed using Thermo Scientific Proteome Discoverer 2.4 software and the SEQUEST^®^ HT search engine. The data were searched against a Mus musculus fastafile with a 1% false discovery rate (FDR) using Percolator. Further processing was performed using a new Rt-Aligner and created Feature Mapper nodes for the untargeted label-free quantification workflow. FDRs for proteins, peptides, and peptide spectral matches were kept at 1%. All results were filtered by a q-value of <0.01 (equals an FDR of 1% on the peptide level and a filter of a minimum of 2 unique peptides). The presented data are from three sets of independent experiments, and from each set, three replicates were processed to control for intra- and inter-assay errors.

### 2.12. Bioinformatics Analyses

RNA-Seq downstream analysis was performed using in-house R scripts. The volcano plot and heatmap were generated using R packages ggplot2 and heatmap, respectively. Functional classification and enrichment analysis were based on GO annotation and the Kyoto Encyclopedia of Genes and Genomes (KEGG) database [25]. To detect the pathways differentially perturbed in verapamil-treated cells as compared to untreated cells, a computational method was used to integrate differential gene expression into predefined pathways, as previously described [32]. Briefly, P = (G, I) was graphed, where “P” is the pathway, “G” is their gene sets, and “I “depicts the interactions between these genes. The fold change (treatment vs. control) data were entered, and the pathways perturbed by verapamil treatment were identified. The Liptak–Stouffer z-score was calculated as the perturbation of each subgraph, and the most perturbed subpathway was computed using the algorithm, as described elsewhere [32]. Bonferroni corrections were applied to compute *p*-values of the most perturbed pathways for more stringent proteomic and transcriptomic results.

### 2.13. In Vivo Study Using Transgenic Zebrafish Embryos Model

Zebrafish (Danio rerio) were reared at 28 °C under a 12 h light/dark cycle at the Dasman Diabetes Institute’s Animal Facility. The fish were bred as per the guidelines approved by the Animal Care Ethical Committee (RA-2019-005) in accordance with the National Institutes of Health (NIH) guide for the care and use of laboratory animals (NIH Publications No. 8023, revised 1978) and were in compliance with the ARRIVE guidelines and the standard laboratory procedures for zebrafish [33]. Ins:NfsB-mCherry transgenic zebrafish was a generous gift from Dr. Michael J. Parsons, University of California, Irvine, USA. In this ins:NfsB-mCherry transgenic model, the bacterial gene encoding the nitro-reductase (NTR) enzyme, which converts a prodrug such as metronidazole (MTZ) to cytotoxins was fused to an mCherry fluorescent reporter under the control of an insulin proximal promoter [34]. MTZ (M3761, Sigma, St. Louis, Mo, USA) was dissolved in E3 media, as previously described [35], and verapamil was dissolved in deionized water. Embryos were reared in E3 media at 28 °C in the dark, and to study the β-cell protective effect of verapamil, larvae were monitored 3–6 days post fertilization (dpf) for β-cell regeneration by detecting mCherry fluorescence intensity. The transgenic larvae were grouped as follows: group 1, control (untreated larvae); group 2, larvae treated with 10 µM verapamil; group 3, larvae treated with 10 µM verapamil for 24 h and then exposed to fresh E3 media containing 10 mM MTZ for 48 h; and group 4, embryos treated with 10 mM MTZ at 4 dpf for 48 h. Full body images (75X magnification; 200 ms exposure time) were acquired using a Discovery V12 Stereo microscope (Zeiss, Jena, Germany) and an Alexa Flour 594 red fluorescent emission filter.

### 2.14. Statistical Analysis

The data obtained were expressed as mean ± SEM values from at least three individual experiments. The significance of group differences was calculated using an unpaired Student’s *t*-test or one-/two-way analysis of variance (ANOVA) test, followed by Tukey’s multiple comparisons test using GraphPad Prism ver. 9.0 (GraphPad Software, Boston, MA, USA). *p*-values <0.05 were considered statistically significant and expressed as * *p <* 0.05, ** *p <* 0.01, *** *p <* 0.001, or **** *p <* 0.0001.

## 3. Results

### 3.1. Verapamil Induces Proliferation of MIN6 Cells

**Figure 1:**
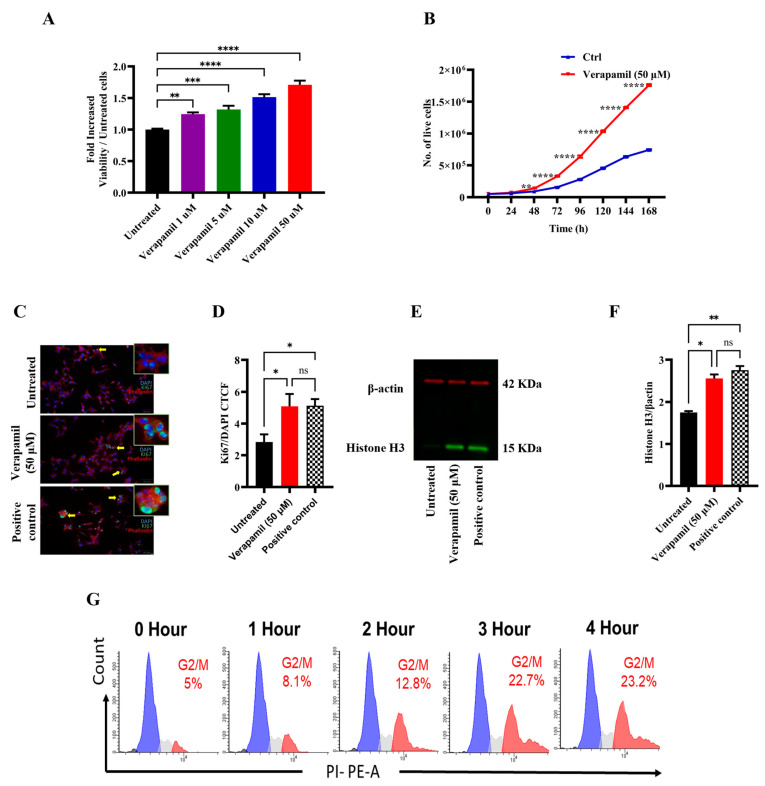
Proliferative effect of verapamil in MIN6 cells. (**A**) MIN6 cells were maintained in 5.6 mM glucose and were treated with different concentrations (1, 5, 10, and 50 μM) of verapamil for 24 h. (**B**) Growth curve of MIN6 cells cultured in 5.6 mM glucose media and treated with verapamil (50 μM, red line) in comparison with untreated cells (blue line). (**C**,**D**) Ki67 analysis showing the influence of verapamil on cell proliferation. MIN6 cells were cultured in serum-free conditions in the presence or absence of verapamil. Cells cytoskeletons were labeled with phalloidin (red) and the proliferation level was monitored with the expression of KI67 protein (green). Yellow arrows are presenting the cells expressing Ki67 in the nucleus (cyan color). DAPI was used to counterstain the nucleus. Scale bar = 50 μM, n = 2. The corrected total cell fluorescence (CTCF) was calculated from 10 different fields of each (n) number. (**E**,**F**) Western blot analysis of histone H3 expression corrected to β-actin with representative immunoblots, n = 2. Flow cytometry analysis was conducted to investigate the influence of verapamil induction in a time-dependent manner. (**G**) Histograms of the cell cycle phases. n = 4. The black, blue, gray, and red color histograms are representative of the percentage of the dead cells and the cells in G0/G1, S, and G2/M cell cycle phases, respectively. Data are presented as mean ± SEM values and were analyzed using one-way ANOVA with Tukey’s multiple comparisons test. ns: non-significant, * *p* ≤ 0.05, ** *p* ≤ 0.01, *** *p* ≤ 0.001, **** *p* ≤ 0.0001 versus control.

First, we studied the effect of verapamil on MIN6 β-cell proliferation using an MTT assay, and the data show a significant, dose-dependent increase in cell proliferation in response to verapamil treatments (1, 5, 10, and 50 µM), as compared to the control (untreated cells) (Figure 1A). The growth curve of MIN6 cells treated with verapamil (50 µM) vs. the untreated control shows a significantly increased proliferation rate (Figure 1B). We also demonstrated that β-cell proliferation was increased by verapamil under serum-starved conditions. In this regard, Ki67 expression (cyan) was significantly enhanced following treatment with verapamil (50 µM), as compared to the control, further indicating an increase in β-cell proliferation (Figure 1C,D). In parallel, verapamil-treated cells also showed increased protein expression of phosphorylated histone H3 (PHH3), a specific mitosis and proliferation marker, compared to the untreated control (Figure 1E,F). 

We then asked if this effect of verapamil on β-cell proliferation was time-dependent. To this end, serum-starved MIN6 cells were treated with verapamil for 1, 2, 3, and 4 h, followed by cell cycle analysis. Verapamil treatment significantly increased the proportion of cells undergoing mitosis, as shown by the G2/M phase expansion. Of note, this effect was time-dependent, and the longer the cells were incubated with verapamil, the higher the number of cells detected in the G2/M phase (Figure 1G). Together, these data establish that verapamil increases MIN6 β-cell proliferation in a time-dependent manner. 

### 3.2. Verapamil Pretreatment Protects MIN6 β-Cells from Cytotoxic Insults Induced by STZ and T1D-/T2D-Cytomixes 

**Figure 2:**
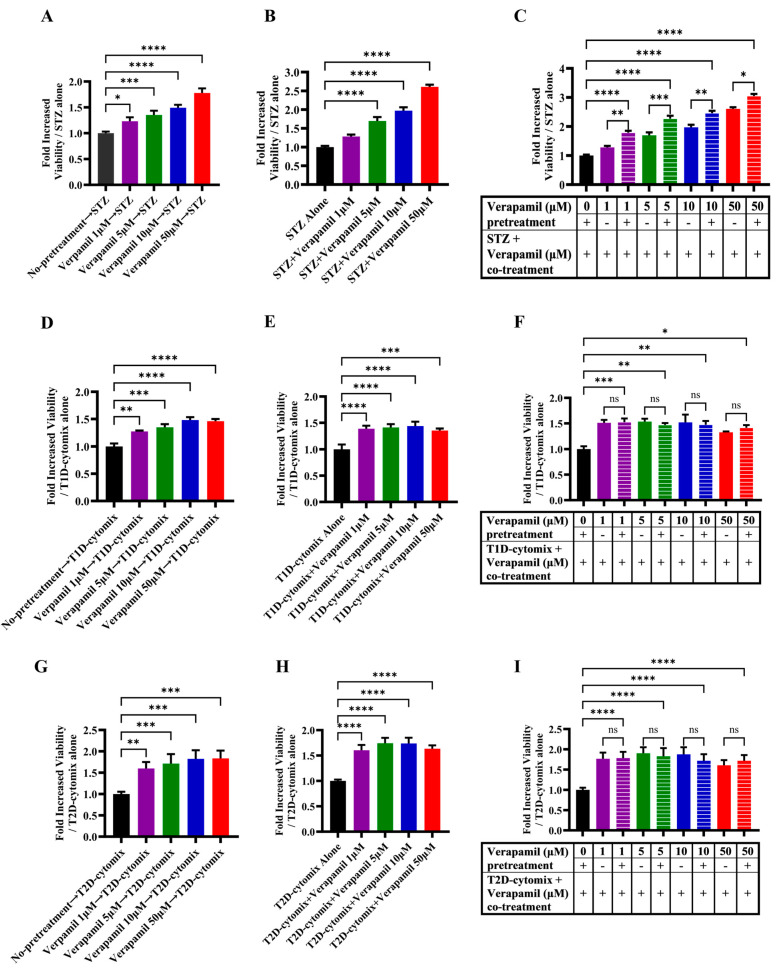
Protective effect of verapamil in MIN6 cells exposed to STZ, T1D-cytomix, or T2D-cytomix stressors. Bar graphs presenting MTT assay results as a ratio of each treatment over viability of MIN6 cells maintained in 5.6 mM glucose. (**A**–**C**) Cells were stressed with streptozotocin (STZ). (**A**) Cells were pretreated with different concentrations (1, 5, 10, and 50 μM) of verapamil for 24 h, the media were changed, and cells were stressed by 3 mM of STZ for another 24 h. (**B**) Cells were treated with a combination of STZ (3 mM) and verapamil (1, 5, 10, and 50 μM) for 24 h. (**C**) Cells were pretreated with verapamil (1, 5, 10, and 50 μM) for 24 h, then fresh media containing both STZ (3 mM) and verapamil (1, 5, 10, and 50 μM) were supplied to the cells for another 24 h. (**D**–**F**) Cells were stressed with T1D-cytomix (IL-1β: 50 ng/mL, TNF-α: 50 ng/mL, and INF-γ: 100 ng/mL) in each setting independently. (**D**) Cells were pretreated with different concentrations (1, 5, 10, and 50 μM) of verapamil for 24 h, then media were changed, and cells were stressed with T1D-cytomix for another 24 h. (**E**) Cells were treated with a combination of T1D-cytomix and verapamil (1, 5, 10, and 50 μM) for 24 h. (**F**) Cells were pretreated with verapamil (1, 5, 10, and 50 μM) for 24 h, then fresh media containing both T1D-cytomix and verapamil (1, 5, 10, and 50 μM) were supplied to the cells for another 24 h. (**G**–**I**) Bar graphs presenting MTT assay results as a ratio of each treatment over the viability of cells stressed by T2D-cytomix (IL-1β: 50 ng/mL, TNF-α: 50 ng/mL, and Palmitic Acid: 500 μM) in each setting independently. (**G**) Cells were pretreated with different concentrations (1, 5, 10, and 50 μM) of verapamil for 24 h, then media were changed, and cells were stressed with T2D-cytomix for another 24 h. (**H**) Cells were treated with a combination of T2D-cytomix and different concentrations (1, 5, 10, and 50 μM) of verapamil for 24 h. (**I**) Cells were pretreated with different concentrations (1, 5, 10, and 50 μM) of verapamil for 24 h, then fresh media containing both T2D-cytomix and different concentrations (1, 5, 10, and 50 μM) of verapamil were supplied to the cells for another 24 h. Each experiment was performed by at least five independent repeats. The difference between the groups was statistically analyzed using two-way analysis of variance (ANOVA), and *p* values < 0.05 were considered significant difference. Data are presented as mean ± SEM, * *p*-value < 0.05, ** *p*-value < 0.01, *** *p*-value < 0.001, and **** *p*-value < 0.0001. ns: non-significant.

We next asked whether verapamil was also cytoprotective in addition to promoting the proliferation of MIN6 β-cells. To determine this, cells were either treated with verapamil or left untreated for 24 h, followed by treatment with STZ (3 mM) for another 24 h. As compared to cells treated with STZ alone (control), verapamil-treated cells showed significantly increased viability in a dose-dependent manner (Figure 2A). Next, MIN6 cells were cotreated (STZ plus verapamil) for 24 h, and as expected, the cells exposed to verapamil (except 1 μM concentration) showed significantly increased viability compared to cells treated with STZ alone (Figure 2B). To determine if pretreatment had a boosting effect on β-cell viability, cells pretreated with verapamil were exposed to a combined challenge with STZ and verapamil for 24 h. The cells pretreated with verapamil demonstrated a significantly higher viability compared to cells with no verapamil pretreatment (Figure 2C). Together, these data support that verapamil has a cytoprotective effect against STZ toxicity, and pretreatment with verapamil offers better protection.

Given that the increased expression of IL-1β, TNF-α, INF-γ, and their combined effect results in an enhanced vulnerability of pancreatic β-cells to autoimmune destruction [36], we asked whether verapamil exposure could protect the MIN6 β-cells from cytotoxic effects of these cytokines in our T1D-Cytomix model. To this end, MIN6 β-cells were pretreated with verapamil or left untreated (control) for 24 h, followed by a 24 h challenge with T1D-cytomix containing IL-1β (50 ng/mL), TNF-α (50 ng/mL), and INF-γ (100 ng/mL) to mimic the T1D pancreatic cytokine microenvironment in a clinical setting, and cell viability was determined using an MTT assay. We found that the cells pretreated with verapamil exhibited a significantly increased viability in a dose-dependent manner, as compared to the control (Figure 2D). Similarly, the cells cotreated with verapamil plus T1D-cytomix showed significantly higher viability, as compared to the control (Figure 2E). Next, cells were pretreated with verapamil for 24 h and then challenged by verapamil/T1D-cytomix cotreatment, and no significant differences in cell viability were observed between two differentially verapamil-treated groups; however, both verapamil-treated groups had significantly higher cell viability, as compared to the control (Figure 2F).

Low-grade chronic inflammation induced by obesity is a major risk factor for T2D [37]. We further asked whether verapamil also had β-cell cytoprotective effects against the stressors that mimicked the T2D-associated pancreatic microenvironment. To this effect, MIN6 β-cells were treated with verapamil or left untreated (control) for 24 h and then challenged by a T2D-cytomix containing IL-1β (50 ng/mL), TNF-α (50 ng/mL), and palmitic acid (500 µM) to mimic the T2D microenvironment. After 24 h, cell viability was assessed using an MTT assay. As expected, the cells pretreated with verapamil showed significantly increased viability, as compared to the control (Figure 2G). Likewise, cells cotreated with verapamil and T2D-cytomix also showed significantly increased viability, as compared to the control (Figure 2H). Moreover, no significant difference in cell viability was observed between verapamil pretreated plus cotreated and pretreated only groups, while both groups had significantly higher viability, as compared to the verapamil-untreated control (Figure 2I). 

### 3.3. Verapamil Enhances Pancreatic β-Cell Function and Glucose Sensing in MIN6 Cells

**Figure 3:**
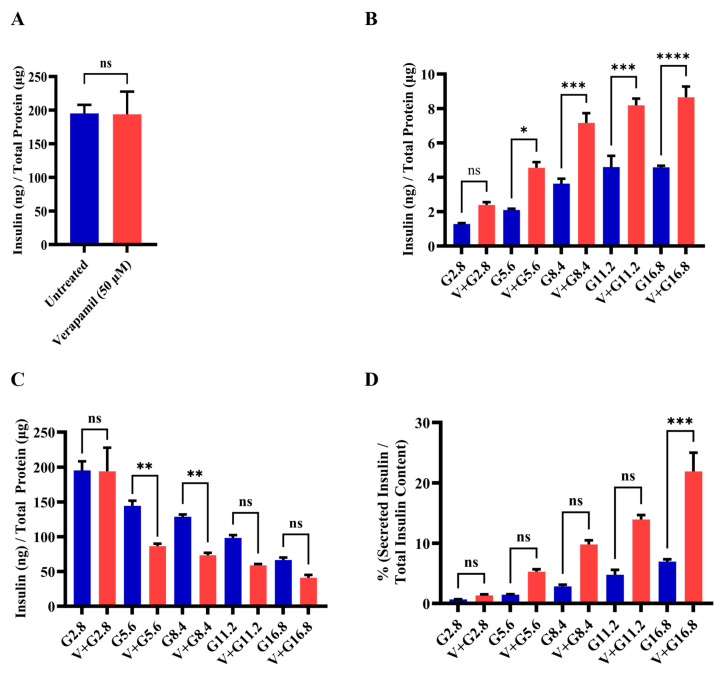
Functional effect of verapamil on the insulin content and glucose stimulated-insulin secretion (GSIS) rate of MIN6 cells. (**A**) Measured insulin content in MIN6 cells treated with 50 µM verapamil (red) for 24 h or left untreated (blue). (**B**) Total secreted insulin by MIN6 cells and (**C**) total insulin content in MIN6 cells when stimulated with different concentrations of glucose and either treated with 50 µM verapamil (red) for 24 h or left untreated (blue). (**D**) Percentage of secreted insulin over total insulin content of each condition. Each experiment was performed in 4 independent repeats. Difference between the groups was statistically analyzed using two-way analysis of variance (ANOVA), and *p*-values < 0.05 were considered statistically significant. Data are presented as mean ± SEM; * *p*-value < 0.05; ** *p*-value < 0.01; *** *p*-value < 0.001; and **** *p*-value < 0.0001, ns: non-significant.

To determine whether verapamil affects insulin production and the function of MIN6 β-cells, total insulin content was compared in verapamil-treated (50 µM) and untreated (control) cells. To this end, no significant differences were found between the two conditions (Figure 3A). Next, MIN6 β-cells were stimulated with different glucose concentrations to determine the GSIS response to verapamil (50 µM) for 24 h, compared to the untreated control. The data show that the GSIS was significantly enhanced (*p* < 0.05) in verapamil-treated cells exposed to 5.6, 8.4, 11.2, and 16.8 mM glucose, as compared to the control (Figure 3B). Measuring insulin content in parallel, verapamil-untreated cells showed significantly higher (*p* < 0.05) insulin content after stimulation with 5.6 mM and 8.4 mM of glucose, as compared to verapamil-treated cells (Figure 3C). Based on the above findings, we further aimed to determine the glucose-sensing capability and function of MIN6 β-cells by calculating the percentage of secreted insulin over total insulin content. The results show that verapamil-treated MIN6 β-cells were relatively more sensitized to glucose stimulation at designated glucose concentrations, and the percentages of secreted insulin over total insulin content differed significantly between verapamil-treated and untreated β-cells under a high glucose challenge (16.8 mM; *p* < 0.01) (Figure 3D). These findings indicate that verapamil enhances β-cells glucose-sensing and insulin-secretion capabilities.

### 3.4. Verapamil Treatment Promotes Mitochondrial Respiration in MIN6 β-Cells 

**Figure 4:**
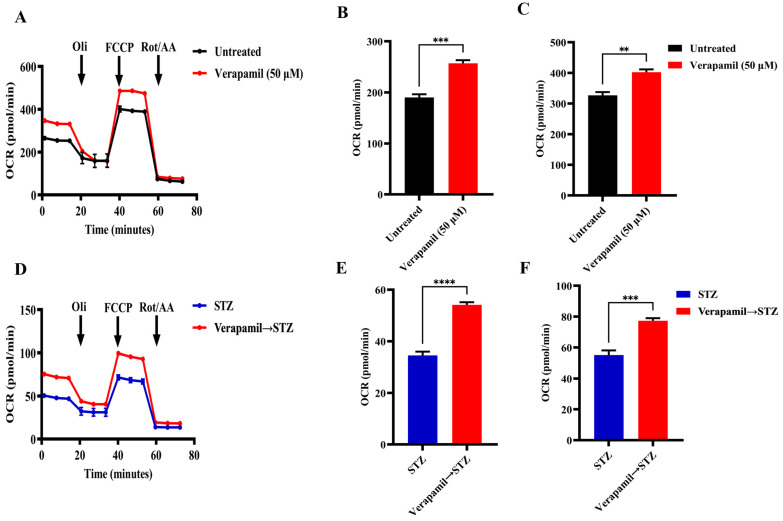
Verapamil pretreatment increases mitochondrial respiration in MIN6 cells treated with streptozotocin. MIN6 cells (4 × 10^4^) were cultured in 5.6 mM glucose media and treated overnight with verapamil (50 µM) or were left untreated. Cells were subsequently treated with streptozotocin (STZ, 3 mM) or left untreated and subjected to metabolic flux analysis. (**A**) Metabolic flux analysis measured oxygen consumption as cells were treated with Oligomycin (1 µM), Carbonyl cyanide 4-(trifluoromethoxy)phenylhydrazone (FCCP, 2 µM), and Rotenone/Antimycin A (Rot/AA, 0.5 µM). (**B**) Basal respiration in metabolic flux analysis before Oli administration. (**C**) Maximal respiration in metabolic flux analysis before Rot/AA administration. (**D**) Metabolic flux analysis of cells pretreated with verapamil prior to STZ treatment. (**E**) Basal respiration and (**F**) maximal respiration in metabolic flux analysis of cells pretreated with verapamil. Reported OCR was normalized to the extracted total protein as an indirect estimation of the number of cells subjected to metabolic flux analysis. Difference between the groups was statistically analyzed using two-way analysis of variance (ANOVA), and *p*-values < 0.05 were considered statistically significant, n = 4 per group, ** *p*-value < 0.01; *** *p*-value < 0.001; and **** *p*-value < 0.0001.

Given the critical role of mitochondrial function in insulin secretion, we next proceeded to investigate mitochondrial respiration in MIN6 β-cells, following treatment with verapamil. To this end, MIN6 β-cells were cultured in a medium containing 5.6 mM glucose and were either treated with verapamil (50 µM) for 24 h or left untreated (control) and were later subjected to metabolic flux analysis. In metabolic flux analysis, β-cells treated with verapamil showed significantly increased oxygen consumption (Figure 4A), including basal (Figure 4B) and maximal respiration (Figure 4C) levels, as compared to the untreated control. To determine the effect of verapamil pretreatment on mitochondrial respiration in STZ-stressed MIN6 β-cells, the cells were cultured with verapamil (50 µM; 24 h) and subsequently treated with STZ for another 24 h, followed by metabolic flux analysis. We found that verapamil-pretreated cells had a significantly higher oxygen consumption than the verapamil-untreated control challenged with STZ alone (Figure 4D). Moreover, verapamil pretreatment significantly increased both basal respiration (Figure 4E) and maximal respiration (Figure 4F). Altogether, a higher OCR at both the basal and maximal levels in verapamil-pretreated cells indicates that the β-cells are metabolically active, viable, and potentially functional and that the verapamil exposure prior to the STZ challenge might additionally confer a protective effect on MIN6 β-cell functionality. 

### 3.5. Transcriptomic and Proteomic Profiling of Verapamil-Treated MIN6 β-Cells

**Figure 5:**
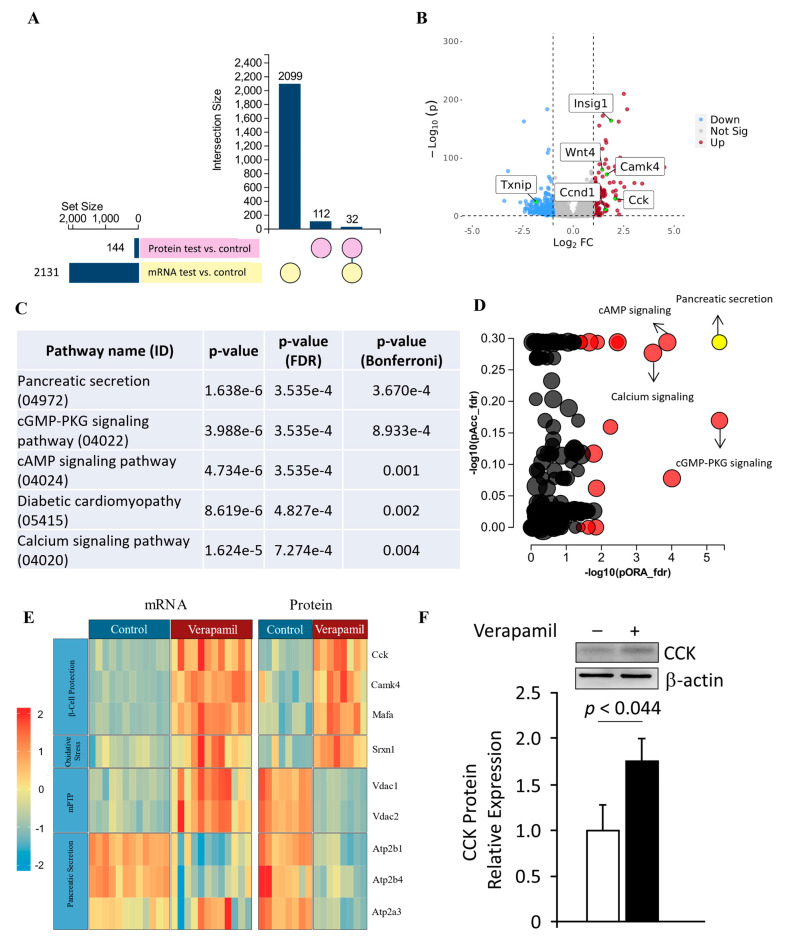
Transcriptomic and proteomic landscape of MIN6 cells treated with verapamil. (**A**) Out of a set size of 2131 genes (yellow) and proteins (pink), 2099 genes and 112 proteins were found to be differentially expressed. Of these, shared differential expressions of 32 genes and proteins were observed. (**B**) Volcano plot showing differentially expressed genes in MIN6 mouse beta cell line treated with verapamil (50 uM) for 24 h (|FC| > 2, *p* < 0.05). Vertical lines are drawn at the |log2FC| thresholds (**C**,**D**) Perturbation vs. over-representation pathway plot: dots representing the top 10 impacted pathways are positioned by their *p*-values from two different analyses: an impact analysis measuring total perturbation accumulation (pAcc) vs. a classical over-representation analysis (pORA). Pathways with significant combined *p*-values are shown in red. The selected pancreatic secretion pathway is shown in yellow. The size of each dot denotes the total number of genes in the corresponding pathway. (**E**) Protein and mRNA levels of key genes found to be up- or down-regulated when exposed to verapamil treatment, along with their respective molecular functions shown in the y axis. Each cell depicts one independent experiment. The expression profiles of the respective genes are depicted by color gradient (*p* < 0.05, |log2FC| > 1). The color scale indicates upregulation in red and downregulation in blue. (**F**) Western blot analysis of CCK expression normalized to β-actin expression; a representative immunoblot is shown.

To further identify the molecular mechanisms underlying the above-mentioned potential benefits of verapamil on MIN6 β-cells, the changes in gene and protein expression profiles after verapamil treatment were studied using RNA sequencing and proteomics, respectively. Comparing verapamil-treated and untreated cells cultured in 5.6 mM glucose milieu, 2131 genes and 114 protein targets were identified, of which 32 targets were differentially expressed at both the gene and protein levels (Figure 5A,B, Table 1). The datasets generated from the current study are available in the GEO repository (GEO accession No. GSE230803). 

We used the impact analysis method [38,39,40] to identify the overrepresented differentially expressed proteins in each pathway and the perturbation in that pathway, as computed by the measured expression across the pathway topology. After FDR and Bonferroni corrections, cardinal signaling pathways were represented in proteomic signatures from our samples, including those associated with pancreatic secretion, cGMP-PKG signaling, cAMP signaling, diabetic cardiomyopathy, and calcium signaling (Figure 5C,D). The most significantly represented pathway, i.e., pancreatic secretion, was further interrogated, and overall pathway perturbation was represented using KEGG resource diagrams (Figure S1A–C). An expression heatmap revealed similar patterns in up- or down-regulation between the gene and protein expression for each target, except for VDAC1 and VDAC2, indicating possible post-transcriptional regulation (Figure 5E). The most upregulated target at both the mRNA and protein levels in response to verapamil treatment was cholecystokinin (CCK), which we further confirmed using Western blot analysis (Figure 5F).

### 3.6. Verapamil Confers Protection from MTZ-Mediated Pancreatic β-Cell Damage in Transgenic Zebrafish Model

**Figure 6:**
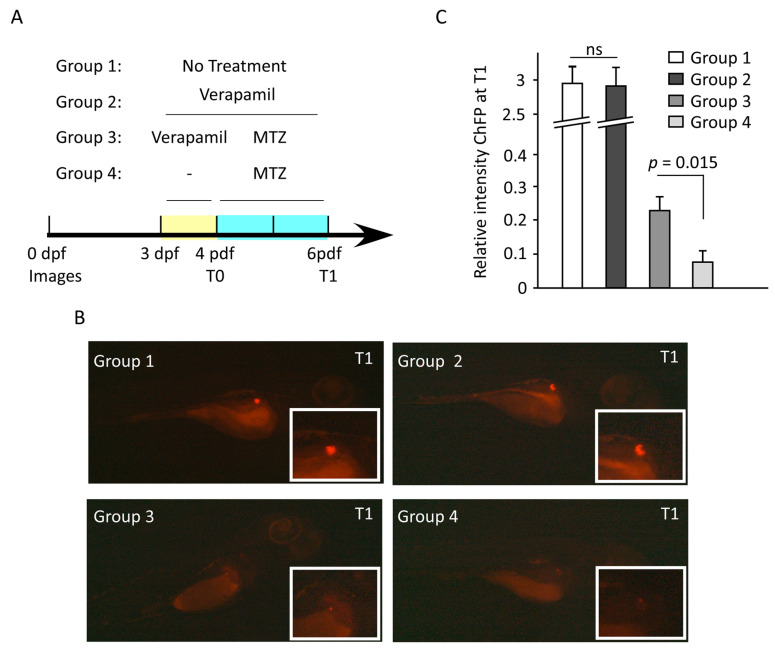
Verapamil pretreatment protects the pancreatic cells against MTZ-induced cytotoxic damage in zebrafish embryos. (**A**) Schematic timeline showing the experimental design and drug treatments. At 3 days post fertilization (dpf), four groups of embryos were treated as follows: Group 1, untreated embryos; Group 2, embryos treated with 10 µM verapamil for 72 h; Group 3, embryos treated with 10 µM verapamil for 24 h, followed by drug removal and administration of 10 mM MTZ for 48 h; Group 4, embryos treated with 10 mM MTZ at 4 dpf for 48 h. Insulin-producing pancreatic cells co-expressing mCherry fluorescence reporter protein (ChFP) were imaged at 4 dpf (T0) and 6 dpf (T1). For each group, the differences in ChFP intensity between T0 and T1 were determined. (**B**) Representative images of pancreatic cells in each group at T1. Inserts depict the magnified ChFP area. Images were taken using Stereo discovery 1.2 ZIESS microcopy. (**C**) Quantification of ChFP intensity in four groups at T1. No significant difference in fluorescence intensity was found between Groups 1 and 2. However, the fluorescence intensity in Group 3, which was pretreated with verapamil, was significantly higher as compared to Group 3, which was not pretreated with verapamil. Experiments were performed in triplicates (n = 20–30 embryos/group). Data represents the mean ± SEM values. ns: non-significant.

Next, we used a transgenic Ins:NfsB-mCherry zebrafish embryo model to study whether verapamil could protect pancreatic β-cells from MTZ-mediated cytotoxic damage. The embryos were divided into the following four groups: Group 1, untreated; Group 2, treated at 3 days post-fertilization (dpf) with verapamil (10 µM) for 72 h; Group 3, treated at 3 dpf with verapamil (10 µM) for 24 h, followed by drug removal and MTZ (10 mM) treatment for 48 h; and Group 4, treated at 4 dpf with MTZ (10 mM) for 48 h. mCherry fluorescence protein (ChFP) fluorescence intensity was detected at 4 dpf (T0) and 6 dpf (T1). We found that verapamil-pretreated embryos (Group 3) showed a significantly higher (*p* = 0.015) fluorescence intensity, as compared to verapamil-untreated embryos (Group 4; Figure 6B,C). This indicates that verapamil induces a protective effect against MTZ cytotoxicity in pancreatic β-cells in the zebrafish model.

## 4. Discussion

Insulin-producing pancreatic β-cells are the key players in glucose homeostasis, and their loss is associated with the development of T1D and the progression of T2D [4,5]. Unsurprisingly, residual β-cell function has been associated with reduced incidence of microvascular complications in both T1D and T2D [41,42]. Currently, promising antidiabetic therapeutic strategies aim at rescuing or preserving the β-cell function and insulin secretory capacity. Herein, we investigated the β-cell cytoprotective and insulinotropic effects of verapamil using T1D- and/or T2D-mimicking stressor challenges in MIN6 β-cells and transgenic zebrafish models. In our MIN6 T1D/T2D stressor models, verapamil treatment improved β-cell viability/proliferation in a dose- and time-dependent manner, as supported by increased expression of Ki67/H3 proliferation markers and evidence of actively replicating β-cells in the G2/M phase. Moreover, in verapamil-treated β-cells, an increase in insulin secretion was observed under high glucose culture conditions. Indeed, this normalized secretion of insulin in verapamil-treated cells was significantly promoted only under high glucose conditions (16.8 mM), compared to lower glucose concentrations tested in our model. It implies that β-cells get hypersensitized by abnormally high glucose levels, a notion which has previously been associated with enhanced blood glucose control and reduction in exogenous insulin requirements [11,13]. Together, these observations lead to the conjecture that verapamil may have a more promising insulinotropic benefit under advanced hyperglycemic conditions.

Notably, while most other studies evaluated verapamil use in diabetic cohorts, we assessed its direct β-cell protective and insulinotropic effects using modified, more clinically relevant models in the setting of verapamil pretreatments or cotreatments, followed by diverse cytotoxic challenges such as T1D-/T2D-cytomixes. The β-cell protective effect was further confirmed at the organismal level using MTZ-mediated β-toxicity in mCherry-reporter zebrafish embryos. We found significantly enhanced β-cell viability in the presence of verapamil in both in vitro and in vivo models, establishing its preventative and β-protective benefits, in line with previous evidence that verapamil lowers the risk of new-onset T2D [3,7,8,11,13,14].

Our analyses of changes in the proteomic and transcriptomic landscapes in verapamil-treated MIN6 β-cells are of particular interest. It is noteworthy that verapamil treatment brought to light a wide array of proteins and genes that were differentially expressed in MIN6 β-cells. Of those, some are remarkable, owing to their involvement in fundamental pathways, ultimately contributing toward maintaining glucose homeostasis and/or preserving pancreatic β-cells. In our transcriptomic analysis, we found *Ccnd1* upregulated at the mRNA level, and the role of this gene in inducing β-cell proliferation is well established [43,44]. In addition, through transcriptome analysis, we also detected the lower expression of the *Txnip* gene in verapamil-treated cells, as compared to untreated cells. TXNIP is a redox regulator protein that was identified as an attractive therapeutic target for restoring β-cell dysfunction, owing to its increased expression in the β-cells of diabetic patients [17,20]. TXNIP overexpression is known to play a key role in β-cell apoptosis, whereas its deficiency promotes endogenous β-cell survival [17,20,45]. Thioredoxin, a protein involved in the antioxidant defense system of β-cells, is inhibited by TXNIP, thereby promoting oxidative stress. Moreover, TXNIP is also associated with inflammasome activity, and its downregulation may have anti-inflammatory effects [46].

In response to verapamil, the most dominantly upregulated factor, at both the mRNA and protein levels, was the glucose-like peptide-1 (GLP-1)-induced incretin—called cholecystokinin (CCK). To the best of our knowledge, we are the first to show that verapamil induces the expression of CCK transcripts and proteins in MIN6 β-cells. CCK plays a role in preserving β-cell mass with increasing age and protecting them against apoptosis induced by STZ and β-toxic proinflammatory cytokines [47]. In our in vitro studies, verapamil induced β-cell protection against insults, including STZ and T1D-/T2D-cytomixes, which could be due, in part, to upregulated CCK expression.

We also found upregulated levels of *Wnt4*, which is a ligand heterogeneously expressed in β-cells, and it plays a central role in activating calcium signaling and lowering blood glucose levels, along with regulating cellular redox homeostasis [48]. Wnt4 is the most abundantly expressed protein in β-cells in high fat, high glucose, or insulin-resistant conditions; it is suggested to play a pivotal role in β-cell proliferation or regulating and enhancing the insulin secretory response induced by the above-mentioned conditions [49]. Kurita et al. suggested that *Wnt4* might possibly be involved in the glucose-induced vesicle transport of insulin in MIN6 β-cells [49]. Moreover, *Wnt4* overexpression was shown to upregulate genes controlling β-cell maturation and function and related to higher mitochondrial abundance [48]. 

An increase in both the mRNA and protein levels of CaMK4 upon verapamil treatment in our study is suggestive of its impact on MIN6 β-cell survival and proliferation. CaMK4, a serine/threonine kinase, has been previously shown to mediate *Irs2* expression in MIN6 cells and mouse islets stimulated by glucose via the activation of the cAMP response element binding protein (CREB) [50]. The present study, based on the previous knowledge that *Irs2* knockout reduces β-cell mass and increases β-cell apoptosis, suggests that CaMK4 may regulate β-cell survival and proliferation, possibly via the IRS2-dependent stimulation of cell proliferation and apoptosis inhibition.

In this study, we found higher levels of basal and maximal mitochondrial respiration in verapamil-treated MIN6 β-cells, which could be attributed to higher *Wnt4* expression, and that, along with reduced TXNIP gene expression, could possibly explain the ability of verapamil to render protection against stressors [15]. Indeed, several studies showed that verapamil treatment reduced the pro-apoptotic TXNIP expression, thereby promoting the survival of β-cells and preventing diabetes [15,20]. Altogether, Wnt4 upregulation by verapamil treatment may have potential as a therapeutic target regarding T2D and, therefore, requires further in-depth studies. 

Mitochondrial dysfunction is related to the pathophysiology of diabetes, resulting in impaired ATP production and defective insulin secretion. VDAC, a key protein found in the outer mitochondrial membrane, regulates the movement of metabolites across the membrane [51] and is a key element in apoptotic signaling [52]. VDAC1 overexpression in various cells has been shown to induce apoptotic cell death [52], and it increased the susceptibility to apoptosis in response to high glucose conditions [53], suggesting its regulatory role as a gatekeeper in mitochondria-mediated apoptosis. *VDAC1* overexpression was found to be driven by impaired glycemic levels, and its inhibition restored GSIS and prevented hyperglycemia in *db/db* mice [54]. Of note, we found that verapamil treatment downregulated protein expression of both VDAC isoforms (VDAC1/2); considering that VDAC1 was upregulated by a state of glucotoxicity over a prolonged period in pre-diabetic patients [53,54,55], verapamil could be an ideal therapy to prevent both the development and progression of diabetes. Taken together, based on growing numbers of studies, we suggest that the anti-diabetic benefit of verapamil could be attributed to: (i) decreased TXNIP mRNA expression [15], (ii) CCK upregulation [47], (iii) Wnt4 upregulation [49], (iv) increased mRNA and protein levels of CaMK4 [56], and/or (v) downregulation of VDAC 1/2 [52]. 

Although our study provides valuable insights into the molecular and cellular mechanisms that are altered by verapamil, it involves certain limitations. Firstly, we have used a single cell line (MIN6 cells), and it would be interesting to also study the effects of verapamil pretreatment on other β-cell models, including the human EndoC-BH1 β-cell-line and primary isolated islets from mice or humans, followed by exposure to stressors treatment, which might have a different impact on these cells, as the cell expression of their respective receptors is different between the species and between primary and tumor cell-lines. Next, the role of genes such as *Wnt4* must be inferred with caution, owing to their heterogeneity and biphasic involvement in both canonical and non-canonical pathways, and mainly due to a lack of deeper understanding of their precise role in diabetes and related disorders. Further studies are required to elucidate the exact role of such factors. 

## 5. Conclusions

Taken together, our data suggest that pretreatment with verapamil may render protection in MIN6 β-cells and zebrafish larvae, following exposure to T1D/T2D mimicking conditions. Characteristic changes identified in the β-cell proteomic and transcriptomic landscapes after verapamil treatment provide valuable insights into the critical molecular reprogramming induced by verapamil, enabling the development of novel strategies targeted at treating diabetes at its root cause, i.e., by resolving β-cell loss or impairment. 

## Figures and Tables

**Table 1:** Up-regulated and down-regulated 32 proteins and their respective genes identified by proteomics/transcriptomics analyses.

Gene Name	logfc Protein (Mass Spec)	adjpv Protein (Mass Spec)	logfc mRNA (RNA-seq)	adjpv mRNA (RNA-seq)
**Acss2**	0.546956178	0.045430822	1.468439926	0.000001
**Ap3m2**	−1.258425153	0.004563029	−0.631083607	0.00535079
**Atad1**	−0.878321443	0.003214256	−0.69090951	0.001886914
**Atl3**	−0.775959726	0.01924926	−0.705494101	0.000782498
**Atp2b1**	−0.768567592	0.001599313	−0.903858193	0.00000179
**Cadm1**	6.64385619	0.000001	−1.111895426	0.000001
**Camk4**	0.618238656	0.025051947	1.667580225	0.000001
**Cck**	1.343692069	0.000001	2.086382128	0.000001
**Fdps**	1.236339539	0.000001	2.06424327	0.000001
**Fn1**	0.750177706	0.00019395	0.707236676	0.000001
**Gabarap**	0.779049553	0.000817148	0.608883531	0.000001
**Gc**	0.575312331	0.010226369	−0.668515132	0.000001
**Gdap1**	−0.744197163	0.03032308	−0.715727824	0.009402028
**Hax1**	6.64385619	0.000001	0.708603816	0.000001
**Hectd1**	1.205392513	0.000001	−0.766523599	0.004572659
**Hmgcs1**	1.608809243	0.000001	1.574403987	0.000001
**Idi1**	1.136191386	0.000001	1.063478538	0.00444946
**Ins2**	−0.706041021	0.003576738	−1.047050788	0.000001
**Kif23**	−0.899695094	0.029438363	−1.278078727	0.000001
**Ldlr**	1.607862903	0.000001	1.459525662	0.000001
**Mafa**	0.715454127	0.019937366	0.86170978	0.000001
**Mid1ip1**	0.82130204	0.000734422	0.902299589	0.000001
**Nsg1**	0.568518598	0.029438363	0.821403563	0.000001
**Pam**	0.600269754	0.008748346	0.772163694	0.000001
**Ppfia2**	−2.53951953	0.000001	−1.455788602	0.000001
**Prlr**	0.619178216	0.031249276	−0.938951004	0.003238752
**Sdf2l1**	0.606915942	0.007338519	1.333480725	0.000001
**Slc25a1**	−0.798366139	0.005415551	0.796013338	0.000001
**Slc9a3r1**	0.508935662	0.025006868	0.846755275	0.000001
**Sqstm1**	0.789103218	8.91352 × 10^−5^	0.605062208	0.000001
**Stc1**	0.804053559	0.000223206	1.114392736	0.000001
**Timm8b**	0.670840336	0.003900441	0.676846573	0.000001

## Data Availability

All data associated with the article are available on request from the corresponding author.

## Supplementary Materials

The following Supporting Information can be downloaded at: https://www.mdpi.com/article/10.3390/cells13110949/s1, Figure S1: Pancreatic secretion (KEGG: 04972); and Supplementary Methods.
