# Supplementary material for: Unraveling Verapamil’s Multidimensional Role in Diabetes Therapy: From β-Cell Regeneration to Cholecystokinin Induction in Zebrafish and MIN6 Cell-Line Models
Source: Cells. 2024 May 30;13(11):949. doi: 10.3390/cells13110949 (PMC11171639; doi:10.3390/cells13110949)
Supplement: Supplementary file 1 [file cells-13-00949-s001.zip › cells-2960413-supplementary.pdf]

Supplementary Methods:

Immunohistochemical Detection of Ki67

To test the pharmacological effect of verapamil on MIN6 cell proliferation, immunohistochemical (IHC) analysis of Ki67 was investigated (22). Cells were seeded on number 1.5 coverslips, 12 mm in diameter, in either low or high glucose media supplemented with 2% bovine serum albumin free fatty acid (BSA FFA) (Cat# 820024, Sigma, St. Louis, MO, USA) for 24 hours. The cells were then either treated with verapamil (50 μM) for another 24 hours or were left untreated. After this culturing period, cells were washed twice with PBS. Then, cells were fixed with 4% paraformaldehyde for 15 minutes at room temperature (RT; 21-23 ^{o}C). Fixed cells were immediately washed twice with PBS and permeabilized in 0.5% saponin for 15 minutes at RT. Next, cells were blocked in blocking solution (5% FBS; 0.1% Tween-20 in PBS) for 40 minutes at RT. Cells were then incubated for 2 hours with rabbit Anti-Mouse Ki67 primary antibodies (Cat# 15580, Abcam, Waltham, MA, USA), diluted 1:1000 in blocking solution, at RT. Next, the cells were washed 3 times, with intervals of 5 minutes, with PBS-Tween (0.1% Tween-20 in PBS), followed by incubation with secondary goat Anti-Rabbit antibody (Alexa Fluor® 488, cat# ab150077, AbCam, Waltham, MA, USA), diluted 1:1000 in blocking solution, and incubated at RT for 1 hour. Then, the cells were also incubated with the Phalloidin-iFluor 594 reagent (Cat#: ab176757, Abcam, Waltham, MA, USA) to visualize their actin filaments. After incubation, the cells were washed 3 times, with 5-minute intervals, with PBS and mounted on the slide with Mounting Medium with DAPI – Aqueous, Fluoroshield (Cat#: ab104139, Abcam, Waltham, MA, USA) to visualize their nuclei. Confocal images were obtained by using inverted Zeiss LSM710 spectral confocal microscope (Carl Zeiss, Gottingen, Germany). All samples were analyzed using the same parameters and the resulting color markup of the analysis was confirmed for each sample. The correlated total cell fluorescence (CTCF) was calculated from 10 different fields of each number (n) *via* ImageJ ver 2.9.0, using the following equation:

CTCF = Integrated Density - (Area of selected cell × Mean fluorescence of background readings) (23).

Western Blotting

Protein extraction and Western Blotting were performed, as previously described (24, 25). Cells were seeded in 6-well plates (Costar, High Wycombe, UK) in either low or high glucose media supplemented with 2% BSA FFA (Cat# 820024, Sigma, St. Louis, MO, USA) for 24 hours. Then, the cells were either treated with verapamil (50 μM) for another 24 hours or were left untreated. After this culturing period, cells were washed with PBS, harvested, and incubated for 30 minutes with lysis buffer (Tris 62.5 mM, 1% Triton X-100, 10% glycerol, pH: 7.5). The lysates were then centrifuged at 14000 × g for 10 minutes, following which, the supernatant was collected. The concentration of protein in lysates was measured using the Quickstart Bradford Dye Reagent, 1x Protein Assay kit (Bio-Rad Laboratories, Hercules, CA, USA). Protein samples (20 or 30 μg) were mixed with sample loading buffer, heated for 5 minutes at 95 °C, and resolved by 12% sodium dodecyl-sulfate polyacrylamide gel electrophoresis (SDS-PAGE). Cellular proteins were transferred to nitrocellulose membrane (Bio-Rad Laboratories, CA, USA) by electroblotting. The membranes were then blocked with 5% non-fat milk in PBS for 1 hour, followed by incubation with primary antibodies Anti-Histone H3 (D1H2) XP® Rabbit mAb #4499 and Anti-β-Actin (8H10D10) Mouse mAb #3700. Both antibodies were purchased from Cell Signaling Technology (Danvers, MA, USA), and CCK (PA5-103116, Abcam, Waltham, MA, USA). The blots were then washed 3 times with TBST and incubated for 2 hours with goat Anti-Rabbit IgG H&L (IRDye® 680RD) (Cat#: ab216777, Abcam, Waltham, MA, USA), goat Anti-Mouse IgG H&L (IRDye® 800RD) (Cat#: ab216772, Abcam, Waltham, MA, USA), or horseradish peroxidase-linked conjugated secondary antibodies. Immunofluorescence or chemiluminescence bands were visualized by Molecular Imager ® ChemiDocTM MP Imaging Systems (Bio-Rad Laboratories, Hercules, CA, USA).

Proteome Sample Preparation

Proteome sample preparation was conducted based on the standard protocol as previously described (31). Frozen cell pellets were lysed according to the Biognosys sample preparation kit protocol (Ki-3013). Cell pellets were lysed with 80 µl of denaturing buffer for 30 minutes at room temperature. A total of 100 µg of total cell lysates were reduced in reduction buffer at 37 °C, by gently shaking for 30 minutes. Samples were then alkylated by adding 16 µL of alkylation solution to each well after cooling, and were incubated at room temperature in the dark for 30 minutes. Samples were then digested by adding 0.4 µg of trypsin at 37°C overnight. Samples were then acidified by adding 20 µl of 10% (v/v) TFA and cleaned-up by using C-18 MACROSpin plates provided by the kit as per protocol. The digested peptides were eluted in 340 µl/well of C18 Elution solution (0.1% (v/v) TFA in 50% Acetonitrile). The samples were then dried down and constituted to a concentration of 1µg/µl and analyzed on the liquid chromatography-electrospray ionization-tandem mass spectrometry (LC-ESI-MS/MS) system.

Samples were analyzed on Q-Exactive HF LC-ESI-MS/MS system coupled with an EASY-nLC™ 1200 nano-LC System through an EASY-Spray Ion Source (Thermo Fisher Scientific, Waltham, MA, USA). Mobile phase A was 0.1% formic acid in water and mobile phase B was 80% acetonitrile plus 0.1% FA in water. LC separation was performed with a trap-elute configuration including a trapping column (Acclaim PepMap100 75 µm x 2 cm) (Thermo Fisher Scientific, Waltham, MA, USA) and an analytical column (ES801A PepMap RSLC C18, 2um, 100A, 50um x 15cm) (Thermo Fisher Scientific, Waltham, MA, USA). The trapping column was loaded by an injection of 5 µg of digested peptide with the mobile phase A delivered at a flow rate of 5 µl/min for 3 minutes to trap and clean peptides, and then the samples were applied onto the column at a flow rate of 300 nl/min. The nano flow column was maintained at 40°C. Peptides were eluted and separated with a 170-minute run. The eluent was ionized using an Easy Spray nano ESI source operating in positive ion mode. Ionization voltage was 2.3 kV and the capillary temperature was 230°C. The Q Exactive HF instrument was operated in the data-dependent acquisition (DDA) mode to automatically switch between full scan MS and MS/MS acquisition. MS1 full scans were acquired for 350-2000 m/z at 120,000 resolution, 60ms maximum IT, 3e6 AGC target. The top 10 multiply charged ions were selected from each scan for MS/MS analysis and then fragmented by higher-energy collisional

dissociation (HCD) with a normalized collision energy setting of 27 eV, at 30,000 resolution, 20 ms maximum IT, 1e5 AGC target, and an isolation window of 2.0 m/z.

Label-free quantitation was performed using Thermo Scientific™ Proteome Discoverer™ 2.4 software using SEQUEST® HT search engine with a precursor mass tolerance of 10 ppm and fragment mass tolerance of 0.02 Da. Enzyme name was set to trypsin, with a maximum missed cleavages value of 2. Carbamidomethylation (+57.021 Da) of cysteine was considered a fixed modification, while oxidation (+15.995 Da) of methionine was considered a dynamic peptide modification. Data were searched against a Mus musculus fastafile with a 1% false discovery rate (FDR) using Percolator. The area of the precursor ion from the identified peptides was calculated using the Feature Mapper and Precursor Ions Quantifier nodes in the Consensus Step and Minora Feature Detector node in the Processing Step with default settings. Further processing was performed using the new Rt-Aligner and Feature Mapper nodes also created for the untargeted label-free quantification workflow in the software. The false discovery rate (FDR) for proteins, peptides and peptide spectral matches (PSMs) were kept at 1%. All results were filtered by a q- value of <0.01 (equals an FDR of 1% on peptide level and a filter of minimum 2 unique peptides.) The obtained data are from three sets of independent experiments and from each set, three replicates were processed to control for intra- and inter-assay errors.

Glucose-Stimulated Insulin Secretion (GSIS) Assay

GSIS assay was performed using the static incubation method (29). Briefly 1×10^5 cells were grown per well, in 24-well plates (Costar, High Wycombe, UK), as a monolayer. After 24 hours, the cells were pretreated with 50 μM verapamil for another 24 hours. The following day, the medium was removed, and cells were washed twice with PBS. Next, for equilibration, the cells were incubated for 1 hour with 500 µl of 2.8 mM glucose, supplemented in Krebs–Ringer HEPES buffer (basal media, pH-adjusted to 7.4 with 1 mol/l NaOH), containing 135 mM NaCl, 3.6 mM KCl, 5 mM $NaHCO_3$, 0.5 mM $MgCl_2$, 1.5 mM $CaCl_2$, 10 mM HEPES, and 0.1% BSA). After equilibration, 500 µl of glucose-containing stimulation media, Krebs–Ringer HEPES buffer supplemented with 2.8, 5.6, 8.4, 11.2, or 16.8 mM glucose, was added to each well and incubated for 2 hours at 37 °C. Following which, the GSIS assay was terminated by placing the plate on ice. Conditioned medium (500 µl) was removed

from each well, centrifuged at 1400 rpm at 4 °C for 5 minutes, was transferred to an ice-cold Eppendorf tube, and was then kept in -20 °C to detect the level of secreted insulin.

To measure the total insulin content of the cells, each well was washed with PBS, after which, 500 µl of cold acidified ethanol (1.5% HCl in 70% EtOH) was added to each tube and kept overnight at -80 °C. The following day, freeze/thaw cycles (-20 °C /4 °C) were performed thrice. The cells were removed by scraping and were transferred to a labeled tube. The tubes were centrifuged for 15 min at 15000 × g at 4 °C. The supernatants were transferred to new labeled tubes, acid-ethanol extracts were neutralized with same volume of 1M Tris pH 7.5, and were kept in -20 °C for insulin measurements using ELISA kit (Mercordia, Sylveniusgatan, Uppsala, Sweden). The levels of total insulin content and secreted insulin were normalized by the level of total protein measured by Bradford assay against a standard curve. All chemicals were purchased form Sigma-Aldrich (St. Louis, MO, USA).

Protection Assays

In this study, MIN6 cells were stressed by one of three stressors, as follows:

1. Streptozotocin (STZ; purchased from Sigma-Aldrich, St. Louis, MO, USA): STZ stock solution (0.3 M) was prepared by dissolving STZ in cold citrate buffer and was used immediately. The final concentration of STZ was 3 mM.
2. T1D-cytomix: A T1D-cytomix was prepared using a combination of TNF-α, IL-1β, and INF-γ (Final concentration: 50, 50, and 100 ng/ml, respectively) (27, 28).
3. T2D-cytomix: A combination of TNF-α, IL-1β, and palmitic acid (Final concentration: 50 ng/ml, 50 ng/ml, and 500 µM, respectively) was used to prepare the T2D-cytomix (27, 28).

   All recombinant cytokines were purchased from R&D systems (Minneapolis, MN, USA).

For the protection assays, the concentrations of verapamil used were 1, 5, 10, or 50 µM, and the protection assays were conducted in the following conditions, as indicated.

1. Pretreatment condition: Cells were pretreated with 1, 5, 10, or 50 μM of verapamil for 24 hours. The next day, the medium was removed, the cells were washed with PBS, and the cells were then exposed to one of the mentioned stressors, as indicated, for another 24 hours. Following which, the MTT assay was performed to measure cell survival.
2. Cotreatment condition: Cells were cotreated with a combination of a stressor and verapamil (1, 5, 10, or 50 μM) for 24 hours, after which, the MTT assay was conducted to measure cell survival.
3. Pretreatment → cotreatment condition: Cells were pretreated with verapamil, as detailed in the "pretreatment" condition, for 24 hours. On the following day, the medium was removed, cells were washed with PBS, and then the "cotreatment" condition, with the indicated stressor, and used verapamil concentration was applied for another 24 hours, and then MTT assay was conducted to measure cell survival.

The MTT assay (Trevigen, Minneapolis, MN, USA) was conducted according to the manufacturer's instructions. The plates were analyzed using the Synergy H4 Hybrid Microplate Reader (BioTek, Winooski, VT, USA), and data analysis was performed using the Gen5 software version 2.03.

Supplementary Figure

Supplementary Figure S1: Pancreatic secretion (KEGG: 04972). (**A**) The overall pathway diagram is overlayed with the computed perturbation of each gene. The perturbation accounts both for the gene's measured fold change and for the accumulated perturbation propagated from any upstream genes (accumulation). The highest perturbation is shown in green. The legend describes the values on the gradient. Note: For legibility, one gene may be represented in multiple places in the diagram, and one box may represent multiple genes in the same gene family. A gene is highlighted in all locations it occurs in the diagram. For each gene family, the color corresponding to the gene with the highest absolute perturbation is displayed in green. **(B)** Represents proteomic and (**C**) mRNA data. The highest negative perturbation is shown in dark blue, while the highest positive perturbation is in dark red.

A

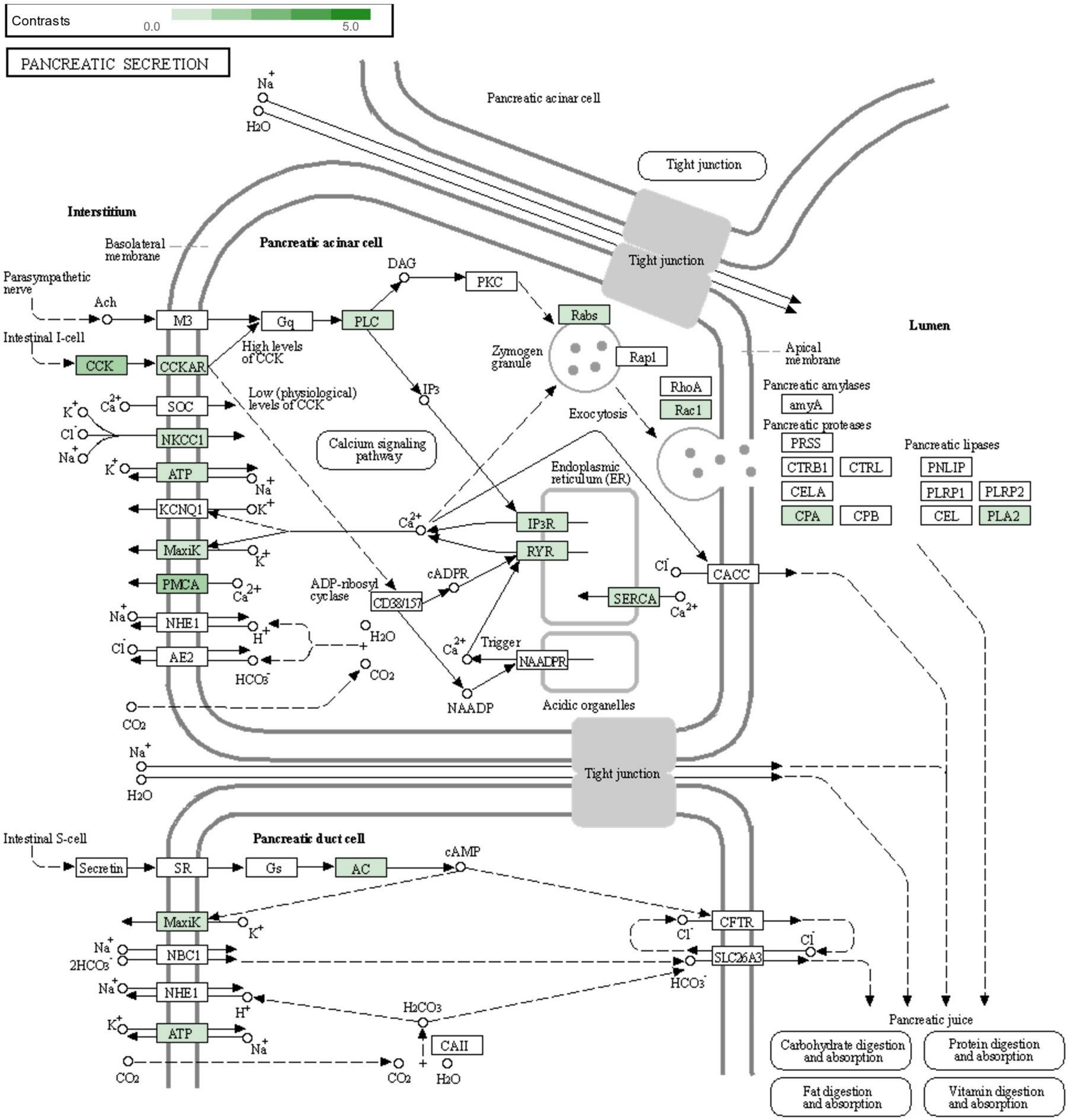

Contrasts
0.0
5.0
PANCREATIC SECRETION
Pancreatic acinar cell
Tight junction
Interstitium
Basolateral membrane
Pancreatic acinar cell
Parasympathetic nerve
Ach
M3
Gq
PLC
DAG
PKC
Rabs
Zymogen granule
Rap1
Lumen
Intestinal I-cell
CCK
CCKAR
High levels of CCK
Low (physiological) levels of CCK
IP3
RhoA
Rac1
Apical membrane
Pancreatic amylases
amyA
Pancreatic proteases
PRSS
CTRB1
CTRL
CELA
CPA
CPB
Pancreatic lipases
PNLIP
PLRP1
PLRP2
CEL
PLA2
SOC
NKCC1
ATP
KCNQ1
MaxiK
PMCA
NHE1
AE2
Calcium signaling pathway
Exocytosis
Endoplasmic reticulum (ER)
IP3R
RYR
SERCA
CACC
ADP-ribosyl cyclase
CD38/157
cADPR
NAADP
NAADPR
Trigger
Acidic organelles
H2O
CO2
HCO3-
Tight junction
Intestinal S-cell
Pancreatic duct cell
Secretin
SR
Gs
AC
cAMP
CFTR
SLC26A3
NBC1
2HCO3-
H2CO3
CAII
Pancreatic juice
Carbohydrate digestion and absorption
Protein digestion and absorption
Fat digestion and absorption
Vitamin digestion and absorption

B

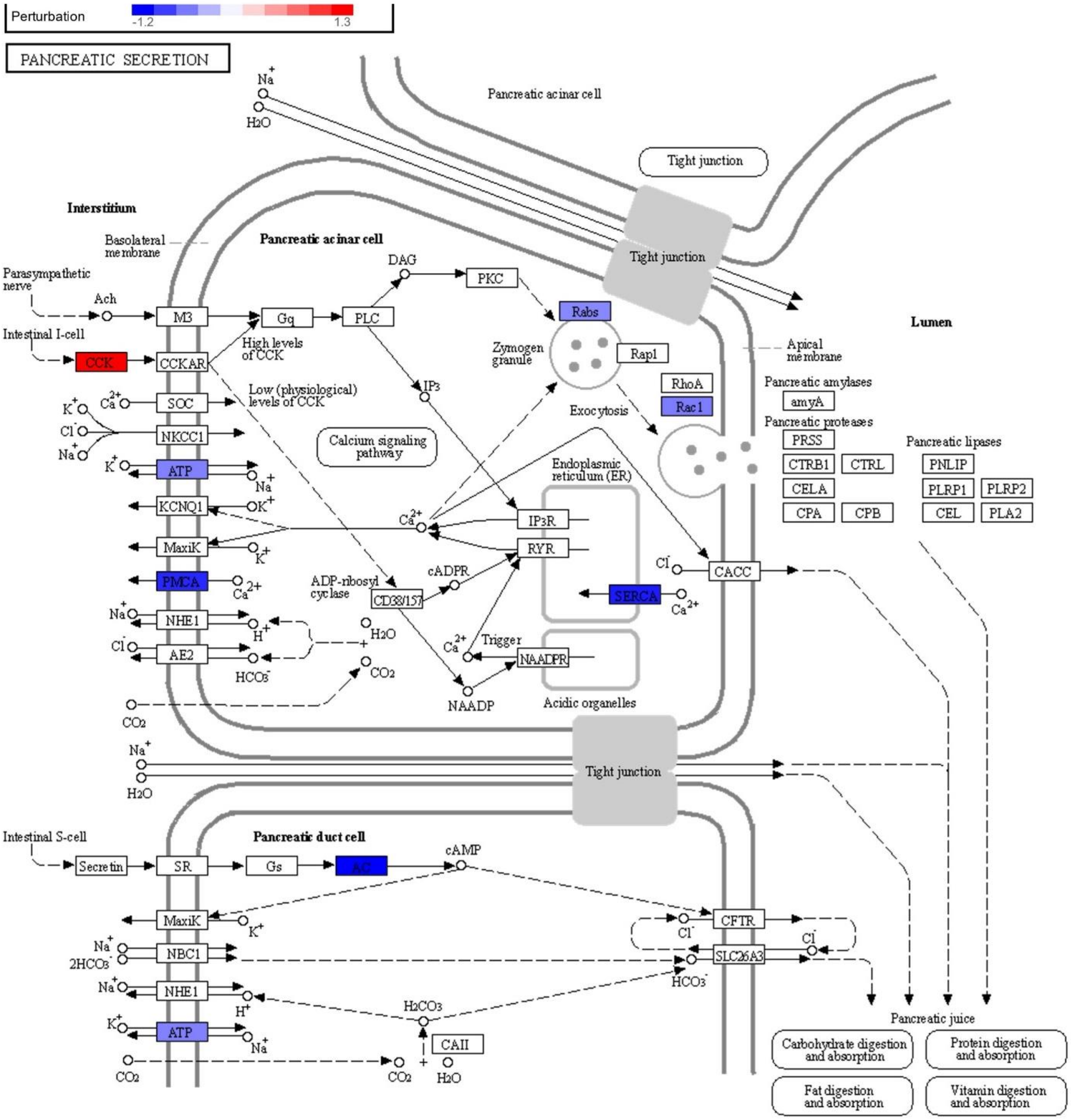

Perturbation
-1.2
1.3
PANCREATIC SECRETION
Pancreatic acinar cell
Tight junction
Interstitium
Basolateral membrane
Pancreatic acinar cell
Parasympathetic nerve
Ach
M3
Gq
PLC
DAG
PKC
Rabs
Zymogen granule
Rap1
Lumen
Apical membrane
Intestinal I-cell
CCK
CCKAR
High levels of CCK
Low (physiological) levels of CCK
SOC
NKCC1
ATP
KCNQ1
MaxiK
PMCA
NHE1
AE2
IP3
Calcium signaling pathway
RhoA
Rac1
Exocytosis
Endoplasmic reticulum (ER)
IP3R
RYR
cADPR
ADP-ribosyl cyclase
CD38/157
SERCA
CACC
Trigger
NAADPR
NAADP
Acidic organelles
Pancreatic amylases
amyA
Pancreatic proteases
PRSS
CTRB1
CTRL
CELA
CPA
CPB
Pancreatic lipases
PNLIP
PLRP1
PLRP2
CEL
PLA2
Intestinal S-cell
Secretin
SR
Gs
AC
cAMP
Pancreatic duct cell
CFTR
SLC26A3
NBC1
H2CO3
CAII
Pancreatic juice
Carbohydrate digestion and absorption
Protein digestion and absorption
Fat digestion and absorption
Vitamin digestion and absorption

C

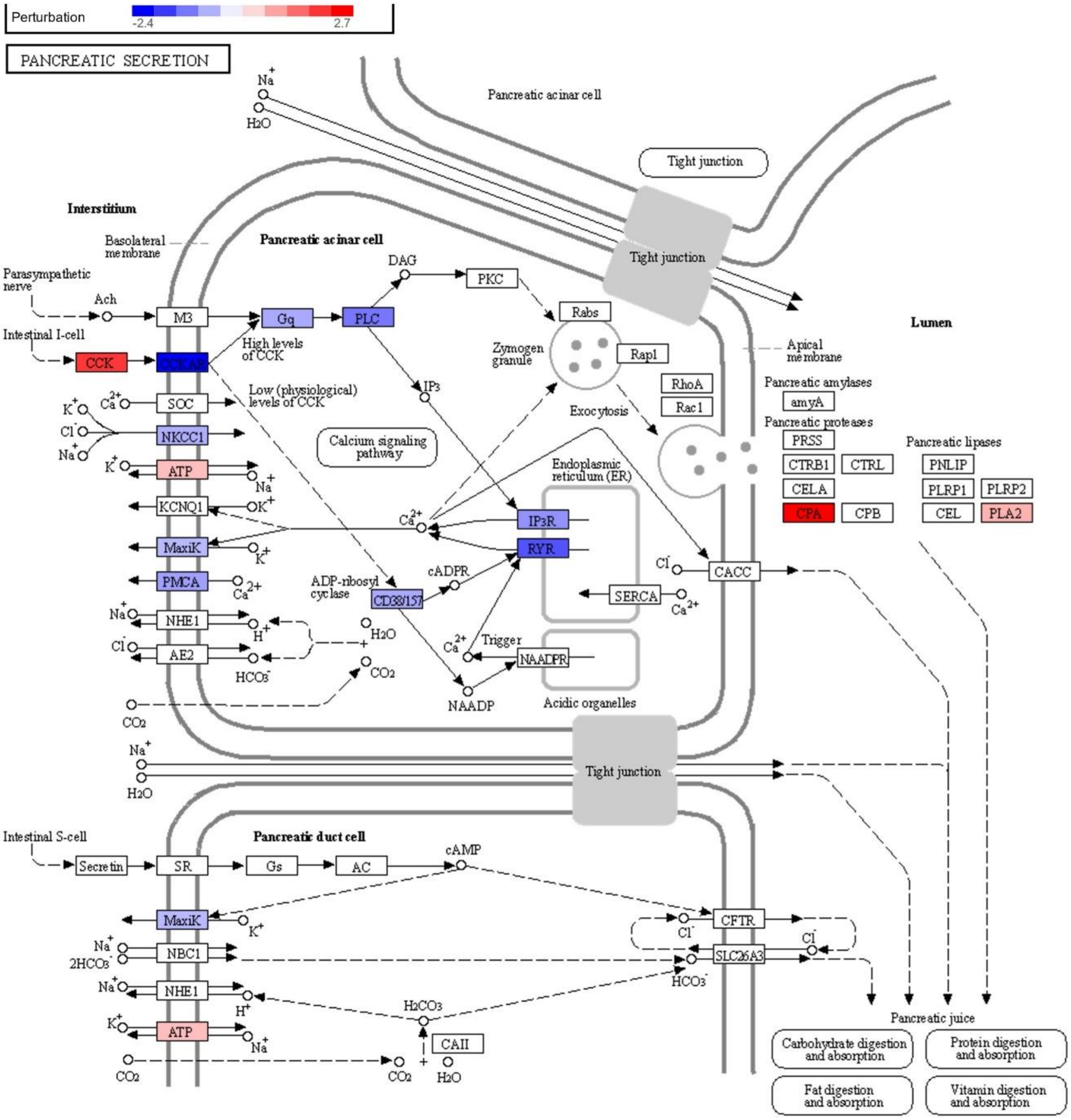

Perturbation
-2.4
2.7
PANCREATIC SECRETION
Pancreatic acinar cell
Tight junction
Interstitium
Basolateral membrane
Pancreatic acinar cell
Parasympathetic nerve
Ach
M3
Gq
PLC
DAG
PKC
Rabs
Intestinal I-cell
CCK
CCKAR
High levels of CCK
Low (physiological) levels of CCK
Zymogen granule
Rap1
RhoA
Rac1
Exocytosis
SOC
NKCC1
ATP
KCNQ1
MaxiK
PMCA
NHE1
AE2
Calcium signaling pathway
IP3
Endoplasmic reticulum (ER)
IP3R
RYR
cADPR
ADP-ribosyl cyclase
CD38/157
SERCA
CACC
Trigger
NAADPR
NAADP
Acidic organelles
Lumen
Apical membrane
Pancreatic amylases
amyA
Pancreatic proteases
PRSS
CTRB1
CTRL
CELA
CPA
CPB
Pancreatic lipases
PNLIP
PLRP1
PLRP2
CEL
PLA2
Intestinal S-cell
Secretin
SR
Gs
AC
cAMP
Pancreatic duct cell
CFTR
SLC26A3
NBC1
CAII
Pancreatic juice
Carbohydrate digestion and absorption
Protein digestion and absorption
Fat digestion and absorption
Vitamin digestion and absorption
